# Supplementary figures and images for: Co-culture models of endothelial cells, macrophages, and vascular smooth muscle cells for the study of the natural history of atherosclerosis
Source: PLoS One. 2023 Jan 20;18(1):e0280385. doi: 10.1371/journal.pone.0280385 (PMC9858056; doi:10.1371/journal.pone.0280385)

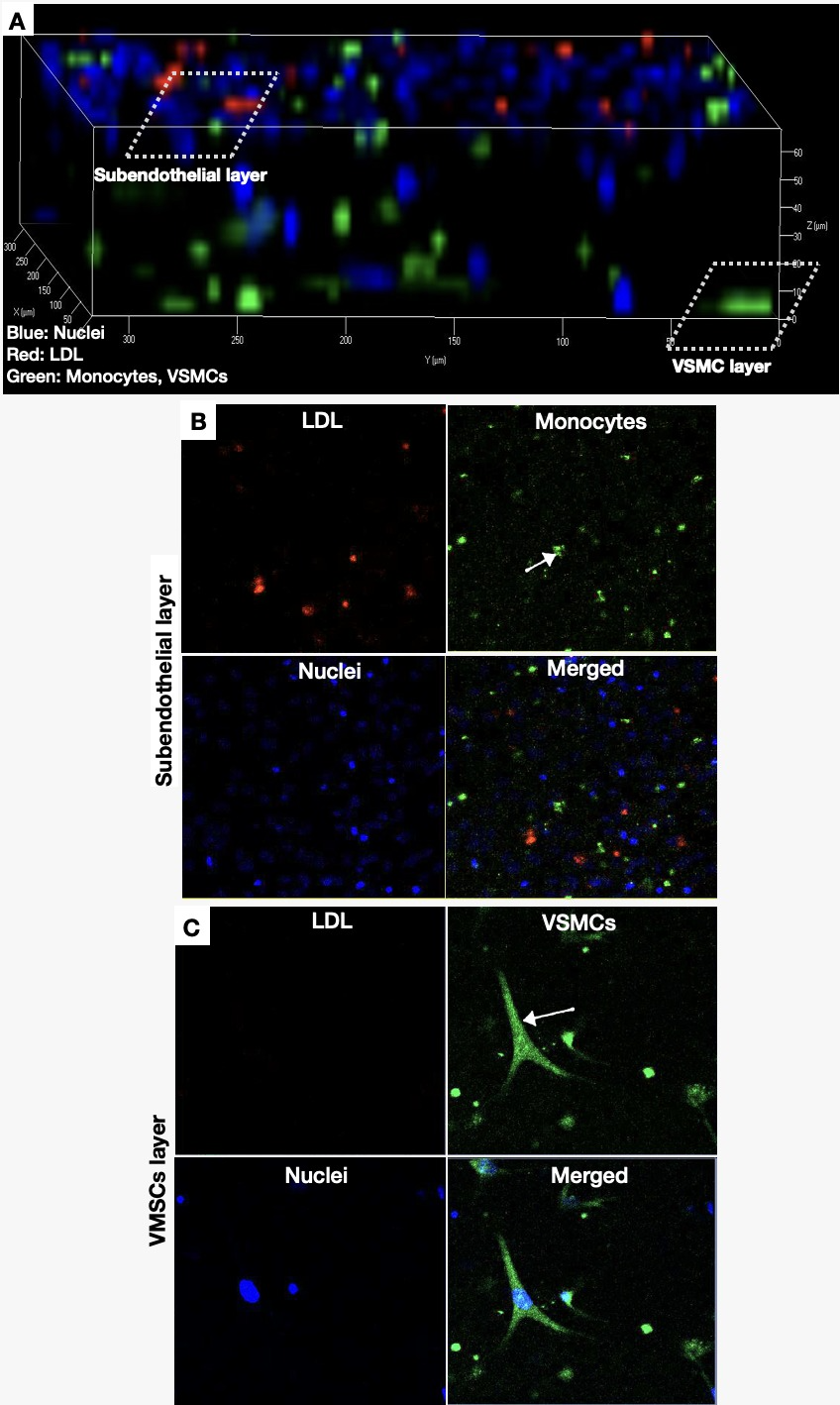

Supplement: S1 Fig — A. Three-dimensional image of the co-culture. Confocal microscope observation on the co-culture of HCASMC, HCAEC, and monocytes from human peripheral blood. B. Images of sub-endothelial layer. Note the Dil-LDL (red), monocytes (green, pre-stained with calcein AM), and endothelial cells (nuclei in blue) at the top of the 3D images. C. Images of smooth muscle cell (SMC) layer. Note the spindle-shaped HCASMC (green, pre-stained with calcein AM) as well as the nuclei (blue) of the cells in the bottom of the 3D co-culture. (TIF) [file pone.0280385.s001.tif]

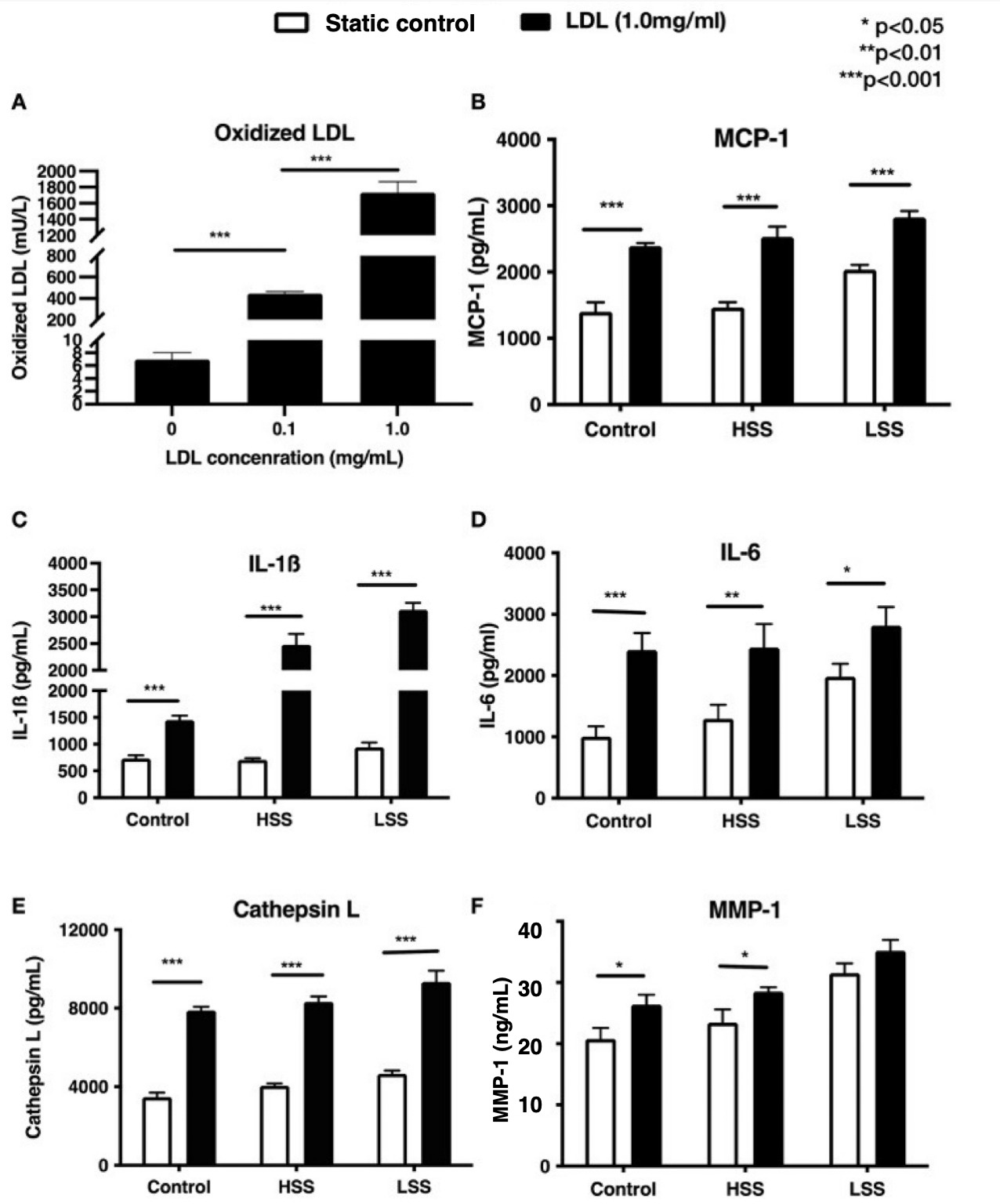

Supplement: S2 Fig — A. Native-LDL concentration-dependent release of oxidized LDL. B-F: Release of MCP-1, IL-1ß, IL-6, cathepsin L, and MMP-1 was increased in cultures exposed to low shear stress, and moreover, in the presence of higher concentrations of LDL, release of pro-inflammatory cytokines and enzymes was further augmented (closed bar). LDL: low-density lipoprotein; MCP-1: monocyte chemoattractant protein- 1; IL: interleukin; MMP: matrix metalloproteinase. *p<0.05; **p<0.01; ***p<0.001. (TIF) [file pone.0280385.s002.tif]

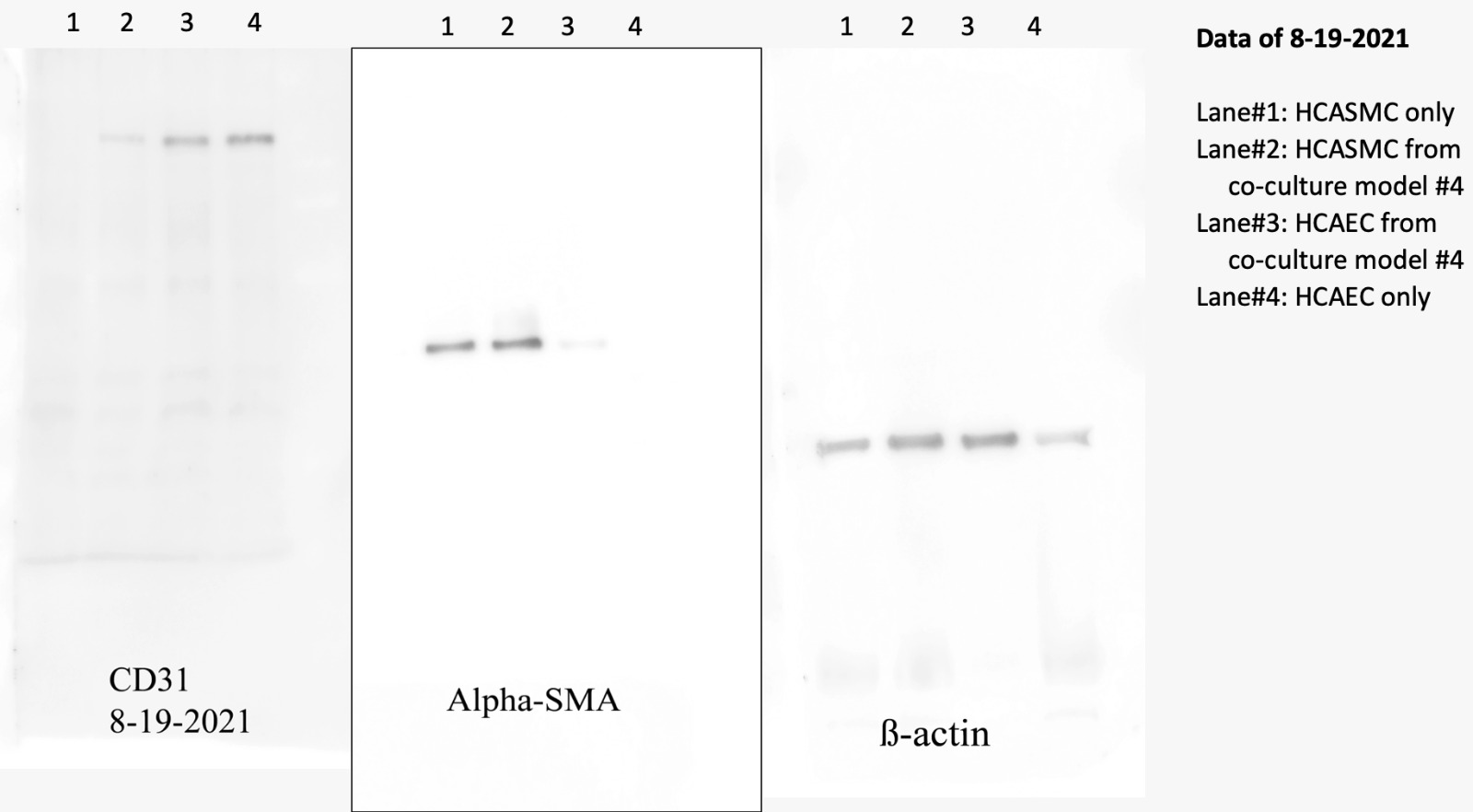

Supplement: S3 Fig — HCAEC: human coronary artery endothelial cell; HCASMC: human coronary artery smooth muscle cell. (TIF) [file pone.0280385.s003.tif]
