# Supplementary material for: Co-culture models of endothelial cells, macrophages, and vascular smooth muscle cells for the study of the natural history of atherosclerosis
Source: PLoS One. 2023 Jan 20;18(1):e0280385. doi: 10.1371/journal.pone.0280385 (PMC9858056; doi:10.1371/journal.pone.0280385)
Supplement: Attachment — Submitted filename: Rebuttal letter_v4.docx [file pone.0280385.s004.docx]

**Reviewer #1:**

In this manuscript by Liu et al, the authors have developed four different co-culture models in order to in vitro replicate different stages of early atherosclerosis: model 1 for normal arterial intima, model 2 for atherogenesis, model 3 for intima xanthomas (mimicking "fatty streaks"), and model 4 for pathological intima thickening. Each model was characterized by electron (SEM and TEM) and confocal microscopies together with measurements of oxidized LDL and expression of chemokines, cytokines and matrix proteinases. Model 4 was also subjected to pre-exposition to low shear stress in order to mimic what is encountered in human atherosclerosis.

Even if the proposed co-culture models suffer from several limitations that are clearly mentioned by the authors at the end of the manuscript (pages 26-27), these models show improvements compared to those already published in the literature and are expected to help researchers working in the field. I recommend acceptation of this work after addressing the following comments.

**Response:** We appreciate the Reviewer’s kind comments.

**Comment 1:** Figure numbering must be corrected. Figure 1 should contain the graphical illustration of the co-culture models and study design but is missing. The current Figure 1 corresponds to the culture model 1 (indicated as Figure 2 in the manuscript). As a result, the numberings of the figures are shifted.

**Response:** We apologize for the missing Figure 1. The error has been fixed in the revised manuscript.

**Comment 2:** Figure relative to model 2: higher magnification/better resolution is recommended for confocal imaging to better show colocalization of LDL particles and endothelial cells.

**Response:** We would like to thank the Reviewer for the suggestion. As shown in panel E of Figure 3, most of the LDL penetrated into the sub-endothelial space and therefore, there was a limited co-localization of LDL with endothelial cells. This is shown evidently in the confocal imaging (panel F) of the same figure. We respectfully believe that a higher magnification of panel F would not make a significant difference to the message conveyed by this picture. However, if the Reviewer insists on the higher magnification panel, we are more than happy to provide it.

**Comment 3:** Figure relative to model 2: what was the concentration of native LDL used? 0.1 and 1.0 mg/dL or 0.1 and 1.0 mg/mL (like for model 3)? This should be confirmed.

**Response:** We apologize for the mislabeling. It was 0.1 and 1.0 mg/mL. This has been corrected in the revised Figure 3H.

**Comment 4:** It would have been interesting to evaluate the permeability of the EC monolayer before and after adding LDL or monocytes?

**Response:** The Reviewer makes an important suggestion. However, unlike the permeability of the EC in monolayer cultures, assessment of the permeability of ECs in the 3D co-culture environment appears to be challenging and remains to be explored in a future study.

**Comment 5:** If comparable concentrations of native LDL were added in the medium for model 2 and model 3 (see previous comment), does it mean that adding monocytes in the model has no influence on the oxidization of native LDL? This should be discussed by the authors.

**Response:** The Reviewer raised an interesting point. Indeed, the comparable amount of oxidized LDL between models #2 and #3 suggests that ECs were the primary source of oxidized LDL. We comment on this issue in the Discussion on pages 23 and 24.

*“Of note, the source of LDL oxidization appeared to be the ECs given that the amount of oxidized LDL between model #2 (ECs only) and #3 (ECs and monocytes) were comparable. This finding is in line with the literature. [4]”*

**Comment 6:** What is the percentage of monocytes that transmigrate through the EC monolayer for models 3 and 4? Is it comparable?

**Response:** This is another interesting point. However, it appears technically very challenging to measure the fraction of monocytes that transmigrate through the EC monolayer given that both the confocal microscopy and electron microscopy captured a small region of the total surface of the 3D culture. We comment on this in the Limitations on page 25.

“*Seventh, we did not perform quantification of monocyte transmigration and transformation into foam cells. This kind of quantified analysis appears to be technically very challenging in the 3D co-culture environment and represents a future investigation.*”

**Comment 7:** Figure relative to model 4: for the western blot profile (panel K), HCASMCs exhibit similar amount of SMA compared to HCASMCs in monoculture. Authors should check other marker proteins in order to better characterize the phenotype of the SMC (contractile versus synthetic). Is there production of ECM by SMC in this model?

**Response:** We would like to thank the Reviewer for this important point. Sophisticated characterization of the SMC phenotype (contractile versus synthetic) is an ongoing project. Here we provide some preliminary data which show that model #4 was capable of producing high amounts of fibronectin suggesting the presence of synthetic SMCs in the culture.

**Comment 8:** Figure relative to model 4: to be homogeneous with figures relative to models 2 and 3, "LDL (µg/mL)" in the legend of panel L should be replaced by "native LDL added in the medium (µg/mL)".

**Response:** Figure 5L has been revised as suggested.

**Comment 9:** Why authors did not used comparable concentrations of native LDL in the medium for model 4 (as for model 2 and 3)? This should be explained by the authors.

**Response:** In model #4, which is a co-culture of all three types of cells, ox-LDL was very high when 0.1 mg/mL native LDL was added into the culture. Even the addition of native LDL at a concentration of 0.05 mg/mL, which was half of the one used in models #2 and #3, resulted in a significantly higher amount of oxidized LDL. The increased amount of oxidized LDL in model #4 was most likely attributed to the multiplying effect of low shear stress. In that context, to keep the amount of oxidized LDL produced by model #4 comparable to the amounts of oxidized LDL produced by models #2 and #3, we elected to use lower concentrations of native LDL in model #4. We comment on this point on page 23.

*“Monocytes were then plated on top of the EC monolayer and allowed to migrate into the subendothelial collagen matrix. Using this model, we examined the effect of LDL and low shear stress on the production of pro-atherogenic molecules, and found that the LDL was oxidized in an LDL concentration-dependent manner (12.5-50 µg/mL native LDL). Notably, oxidation of LDL was dramatically augmented in model #4 (ECs, SMCs and monocytes exposed to low shear stress;* ***Fig 5L****) compared to model #2 (ECs only;* ***Fig 3H****) or model #3 (ECs and monocytes;* ***Fig 4J****), suggesting the multiplying effect of the low shear stress on the LDL oxidation.”*

**Comment 10:** M&M section:
a) page 9: authors indicated that HCAECs and HCASMCs were used between passages 3 and 9. Have the authors checked the phenotype of the cells between passage 3 and 9? Unchanged?
b) page 9: preparation of type I collagen should be better explained. After extraction of collagen, any precipitation and/or dialysis of collagen? What about the presence of HCl?
c) page 12: authors should explain what does the LS column means?

**Response:** a) Regarding the phenotype of the cells between passages 3 and 9, we neither observed any changes in cell shape nor in the expression of biomarkers, such as alpha-smooth muscle actin by HCASMCs, and CD31 or VE-cadherin by HCAECs.

b) Type I collagen was centrifuged after extraction with acid in order to remove non-soluble tissues such as tendon shelf, but it was not precipitated or dialyzed. The HCl was neutralized by mixing with 4 x concentrated DMEM medium (pH 8.5), which results in 1x concentration of DMEM, physiological ionic strength, and pH 7.4.

We comment on this in the manuscript on page 9:

*“Native collagen type I was extracted and purified from rat tail tendon.[11, 12] The tendons were pulled out from rat tails and cut into 1 cm-sized pieces, which were washed three times with tris (hydroxymethyl) aminomethane (Tris)-buffered saline (0.9% NaCl and 10 mM Tris, pH 7.5). Following the wash, the rat tail tendons were dehydrated and sterilized serially with 50%, 75%, 95%, and 100% ethanol. The rat tail tendon collagen was extracted in 6mM HCl at 4°C overnight and purified by centrifuging at 4,000 rpm for 2 hours.* ***The supernatant (pure type I collagen solution in 6mM HCl) was collected and stored at 4°C****.”*

c) LS column is designed for positive selection with the beads by the Miltenyl Biotec. We have revised the manuscript to clarify this on page 11 and 12.

*“HCASMCs were isolated by running through the LS column, which was designed for positive selection of the endothelial cells expressing specific surface biomarker, CD-31 (MACS Milteny Biotec, Auburn, CA, USA).”*

**Comment 11:** page 19: Writing "the LSS effect on the concentration of oxidized LDL in both medium and matrix, as well as the expression of chemokines, cytokines, MMPs and cathepsins was directly proportional to the dose of added native LDL (Fig. 8)" is not correct. For instance, no variation was observed between 5 and 25 µg/mL LDL and between static and flow conditions for Il-1beta, Il-8, CathS, L, MMP1 and MMP9. The authors should modify the text accordingly.

**Response:** We appreciate the Reviewer’s careful reading and kind suggestion. The relevant text has been revised as follows on pages 17 and 18:

*“Notably, the LSS effect on the concentration of oxidized LDL in both medium and matrix, as well as the expression of chemokines, cytokines, MMPs and cathepsins was augmented with increased doses of native LDL* *(****Fig 8A-H****), suggesting the synergistic effect between the pro-atherosclerotic stimulus of low shear stress and LDL.”*

**Comment 12:** Authors should comment on the differences observed for the concentrations measured in the medium+matrix (Fig. 6/7?) versus medium alone (Fig. 7/8?) for the different cytokines, chemokines, MMPs, … at 100µg/mL LDL and under low shear stress. For instance, 150-200 pg/mL MMP-1 in the medium (fig. 7/8?) versus 100.000-150.000 pg/mL in medium+matrix ; around 6.000 pg/mL IL-1b in the medium around 3.000 pg/mL for medium+ matrix.

**Response:** We would like to clarify that the quantification of proteins in Figures 6, 7 and 8 was done in the medium of the cultures and not in the matrix. This has been clarified in the revised manuscript on page 17.

*“Model #4 cultures pre-exposed to low shear stress exhibited significantly higher concentration of oxidized LDL* ***in the medium*** *compared to the cultures pre-exposed to no flow or high shear stress (****Fig 7A****). Similarly, the pre-exposure to low shear stress significantly increased the release of chemokines (MCP-1), cytokines (IL-1ß, IL-6), cathepsins L, and MMP-1,* ***in the medium****, and this effect was augmented by adding LDL* ***in the culture medium*** *(****Fig 7B-F****).”*

Also, we corrected the units to ng/mL in panel F of Figure 7 and panel H of Figure 8.

Fig 7F

Fig 8H

**Reviewer #2:**

The aim of this work is to set up a cell co-culture system to represent the different stages of atherosclerosis. Although the idea of establishing in vitro cell culture system to mimic atherosclerosis is of interest, there are several limitations in this study.

**Comment 1:** in my opinion presenting the different models as representative stages of atherosclerosis development is irrelevant. For example, mixing endothelial cells with collagen is not representative of healthy vessel. They should focus only on their model 4 as in vitro co-culture system to “mimic atherosclerosis”.

**Response:** We appreciate the Reviewer’s kind comments and understand different opinions on our models of “normal” and “atherosclerotic plaque”. However, the advantage of our 3D co-culture model is capable of designing different stages of atherosclerosis development. To clarify, model #1 did not involve a mixture of ECs with collagen. Instead, we covered with ECs a collagen matrix produced with a sophisticated method, and this structure essentially mimics the inner part of the normal arterial wall. In model #2, we added the component of LDL to illustrate the ability of our cultures to represent the early stages of atherosclerosis. In models #3 and #4, we incorporated the monocytes and SMC components coupled with low shear stress to make the whole structure even more representative of the intermediate stages of atherosclerosis. Overall, with all respect to the Reviewer’s viewpoint, we feel that our 3D cultures provide a unique environment to study different stages of atherosclerosis in-vitro.

**Comment 2:** the authors provided images but they should quantify them (e.g. monocyte transmigration, foam cells…) to have an idea of the frequency of the processes

**Response:** In principle, we agree with the Reviewer that the quantification of monocyte transmigration and transformation into foam cells would be helpful. This type of quantifications appears feasible in monocultures. However, they are very technically challenging in our complex 3D co-cultures. In our humble opinion, this kind of quantified analysis of cellular phenomena in 3D cultures goes beyond the scope of our work and would justify a whole new manuscript. However, in our work, we provided qualitative evidence of monocyte transmigration, transformation into foam cells and other key molecular and cellular processes of atherosclerosis. We comment on this in the Limitations on page 25.

“*Seventh, we did not perform quantification of monocyte transmigration and transformation into foam cells. This kind of quantified analysis appears to be technically very challenging in the 3D co-culture environment and represents a future investigation.*”

**Comment 3:** quantifying oxLDL is useless, they should instead visualize oxLDL internalization into the macrophages

**Response:** As suggested, we added a new Figure 6C which shows clearly the co-localization of oxidized LDL with monocytes, macrophages and SMCs in model #4. Also, we provide a comment in the Results on page 15 and Figure 6C legend on pages 15 and 16.

*“To visualize the ox-LDL internalization by monocytes/macrophages, we added exogenous Dil-labeled ox-LDL into the medium of model #4. As shown in Figure 6C, there was a co-localization of oxidized LDL by monocytes/macrophages and SMCs.”*

*“C:* *3D confocal non-immunofluorescence (monocytes and SMCs were pre-stained with calcein AM) image of monocytes/macrophages and SMCs cultured within EC-plated collagen matrix followed by the addition of Dil-labelled ox-LDL (red). Note the co-localization of ox-LDL with SMCs and monocytes/macrophages (M: monocytes; Mø: macrophages; SMC: smooth muscle cells).”*

**Comment 4:** page 22, fig 5 results are missing

**Response:** With all respect, we did not feel that there are missing results in Figure 5.

**Comment 5:** fig 4j, the quantification of oxldl is the same as in fig 3h. In this regard, why adding ldl at 0.1 mg/ml did not give the same result as in fig 2? Anyway, as said before this quantification is useless.

**Response:** A similar comment has been raised by Reviewer #1. We apologize for mislabeling the LDL unit in Fig 3H. It has now been corrected to mg/mL.
