# Supplementary material for: Co-culture models of endothelial cells, macrophages, and vascular smooth muscle cells for the study of the natural history of atherosclerosis
Source: PLoS One. 2023 Jan 20;18(1):e0280385. doi: 10.1371/journal.pone.0280385 (PMC9858056; doi:10.1371/journal.pone.0280385)
Supplement: Attachment — Submitted filename: Rebuttal.docx [file pone.0280385.s005.docx]

**Editor’s comments:**

**Comment 1:** It is stated that the models represent “morphologically and functionally the different histopathologic stages of early atherosclerosis”. It would be more accurate to say that they represent “different cellular mechanisms of early atherosclerosis” as they do not take into account many other cell types and cellular and matrix organization involved in vessel wall during the atherosclerosis process.

**Response:** We very much appreciate the Editor’s kind suggestion. The text has been revised as suggested in the Abstract on page 2:

*“This work aims to present a fast, affordable, and reproducible three-cell co-culture system that could represent* ***the different cellular mechanisms of atherosclerosis****, extending from atherogenesis to pathological intimal thickening.”*

We revised the Introduction on page 5:

*“The aim of this work was to develop a fast, affordable, and reproducible three-cell co-culture model of the atherosclerotic artery wall that could replicate* ***the different cellular mechanisms of atherosclerosis****, extending from atherogenesis to pathological intimal thickening.”*

We revised the Discussion on page 19:

*“In this study, we presented the methodology to construct four different co-culture models of ECs, monocytes/macrophages, and VSMCs, capable of reproducing* ***the different cellular mechanisms of atherosclerosis****, [3] extending from normal intima (culture model #1) to atherogenesis (co-culture model #2), intimal xanthomas (co- culture model #3) and pathological intimal thickening (co-culture model #4).”*

We also revised the Conclusion on page 25:

*“In the current study, we presented a fast, cost-effective, and reproducible co-culture model that can replicate* ***the different cellular mechanisms of early and intermediate stages of atherosclerosis****.”*

**Comment 2:** I understand the challenge of doing an absolute count of macrophages. However, could you provide some relative information on the approximative frequency of observation of macrophages having crossed the endothelial layer in order to indicate to a future user of the models if this is a rare or easy to observe event? For example giving average macrophage number per confocal field you observed / size of observed confocal field.

**Response:** We appreciate the Editor’s kind suggestion. According to our observation on model #3 (Figure 4) and model #4 (Figure 5), we estimated that the number of monocytes that migrated across the endothelial layer into the subendothelial matrix was approximately 5-6 cells per 1,000 monocytes attached on the ECs. We revised the Discussion on page 25:

*“Seventh, in our co-culture models, the quantification of the number of monocytes that transmigrated across the EC layer and infiltrated into the matrix was technically challenging. Our approximate estimation is 5-6 monocytes per 1,000 monocytes attached on the ECs.”*


**Comment 3:** You show that LDL oxidation is strongly increased in model 4 in the presence of SMC in no flow in comparison to model 3. If so, is there a role for SMC in LDL oxidation?

**Response:**  As pointed out by the Editor, the concentration of oxidized LDL was remarkably higher in model 4 (Figure 5L) compared to that in model 3 (Figure 4J), suggesting that the presence of SMC contributes to a significant increase in oxidized LDL. We have revised the Discussion on page 23 accordingly:

*“Also, the oxidation of LDL was increased in model #4 (ECs, SMCs, and monocytes,* ***Fig 5L****) compared to model #3 (ECs and monocytes,* ***Fig 4J****), even under no flow (control) conditions, suggesting that the presence of SMCs plays a role in the augmented LDL oxidization.”*

**Reviewers Comments:**

**Reviewer #1:** (No Response)

**Reviewer #2:** The authors addressed some of my concerns but others remain as following:

**Comment 1:** even that the authors claim that ECs were mixed with collagen in a “sophisticated way “, this does not mean that it is representative of an atherosclerosis stage. In my opinion, this part should be removed from the manuscript as it does not represent an atherosclerotic stage, neither a “healthy vessel”.

**Response:** We appreciate the Reviewer’s suggestion. With all respect, we humbly believe that the addition of ECs on top of the collagen matrix represents as much as possible the structure and function of normal intima. Obviously, the normal arterial wall is way more complex, but our cultures appear to mimic in part the intima.

We discuss this point in the Limitations on page 25:

*“The addition of the ECs on top of collagen matrix appeared to mimic structurally and functionally the normal intima; however, we acknowledge that the normal intima is more complex.”*

**Comment 2:** the authors provided images of monocyte transmigration. I have asked to provide a quantification of this phenomenon, however the authors estimated that this is too challenging. I do not understand the challenging part of this quantification. In case that the infiltration is rare, the authors should acknowledge it.

**Response:** We appreciate the Reviewer’s kind comments. According to our observation on model #3 (Figure 4) and model #4 (Figure 5), we estimated that the number of monocytes migrated across the endothelial layer and macrophages infiltrated into the subendothelial matrix was approximately 5-6 cells per 1,000 monocytes attached on the ECs. We revised the Discussion on page 25:

*“Seventh, in our co-culture models, the quantification of the number of monocytes that transmigrated across the EC layer and infiltrated into the matrix was technically challenging. Our approximate estimation is 5-6 monocytes per 1,000 monocytes attached on the ECs.”*
